# Prognostic-factors for neurodegeneration in chronic moderate-to-severe traumatic brain injury: a systematic review protocol

**DOI:** 10.1186/s13643-020-1281-4

**Published:** 2020-02-03

**Authors:** Bhanu Sharma, Alana Changoor, Leanne Monteiro, Brenda Colella, Robin Green

**Affiliations:** 1 Toronto Rehabilitation Institute, University Health Network, 550 University Avenue, Toronto, ON M5G2A2 Canada; 2 Department of Medical Sciences, McMaster University, 1280 Main Street West, Hamilton, ON L8S 4 L8 Canada; 3 Department of Psychiatry, University of Toronto, 550 University Avenue, Toronto, ON M5G2A2 Canada

**Keywords:** Traumatic brain injury, TBI, Neurodegeneration, MRI, Neuroimaging, Rehabilitation

## Abstract

**Background:**

Traumatic brain injury (TBI) is a leading cause of death and disability. Recently, a paradigm shift in our understanding of moderate-to-severe TBI has led to its reconceptualization as a progressive neurodegenerative disorder. Widespread progressive atrophy is observed in the months and years post-injury, long after the acute effects of the injury have resolved. Some studies have begun to examine prognostic demographic, injury-related, and post-injury risk factors that contribute to these declines. A synthesis of this information, and in particular, an increased understanding of post-injury factors that may be modifiable, would improve our ability to design interventions to reduce neurodegeneration in moderate-to-severe TBI. This systematic review aims to identify prognostic factors for neural deterioration in moderate-to-severe TBI, and thereby inform future intervention research in this population.

**Methods:**

This review protocol was informed by and conducted in accordance with the Preferred Reporting Items for Systematic Reviews and Meta-Analysis Protocols (PRISMA-P) guidelines. Search strategies (designed to identify literature on prognostic factors of neurodegeneration in adults with moderate-to-severe TBI) optimized for MEDLINE, EMBASE PsychINFO, CINAHL, SportDiscus, and Cochrane Central Register of Controlled Trials will be developed with the assistance of a health sciences librarian. Retrieved studies will be screened by two team members. Studies must report on longitudinal neuroimaging (i.e., two or more scans in the same cohort) or neuroimaging in a cross-sectional study and potential prognostic factors for neurodegeneration, such as demographics (e.g., gender, age, education), injury (e.g., severity, etiology), or post-injury characteristics (e.g., type and length of therapy, activity level, mood).

**Discussion:**

By identifying prognostic factors for neurodegeneration, this systematic review can help inform injury management, as well as intervention research designed to offset the effects of modifiable prognostic factors, such as low levels of cognitive or physical activity. In turn, this systematic review can increase our understanding of how to improve outcome following moderate-to-severe TBI.

**Systematic review registration:**

PROSPERO CRD42019122389

## Background

Moderate-to-severe traumatic brain injury (TBI) is a leading cause of death and disability in individuals under the age of 40, and an estimated 1.1% of the general population lives with an ongoing disability resulting from brain injury [1, 2]. The *acute* and *sub-acute* neural consequences of brain injury are well understood, with focal and diffuse mechanical, excitotoxic and hypoxic damage resulting in widespread parenchymal volume losses and compromised white matter integrity [3–6]. Such damage can result in a constellation of cognitive [7, 8], motor [9], and emotional sequelae [10, 11]. Seemingly less well understood, however, are the neural changes that occur in the *chronic* stages of moderate-to-severe TBI.

Our prevailing understanding of the chronic stages of moderate-to-severe TBI has long been one of stability. At a neural level of analysis, it has been assumed that following resolution of acute injuries (e.g., edema, hematoma), brain lesions remain stable thereafter. However, there is now extensive evidence that moderate-to-severe TBI is a progressive and deteriorative disorder. Our lab and others have found extensive evidence of progressive lesion expansion, grey and white matter atrophy, and loss of white matter integrity in the months and years following injury [12–23]. For example, our group observed significant atrophy from 5 to 20 months post-injury in at least one region of interest in 96% of 56 moderate-to-severe brain injury patients studied [13]. Moreover, declines in cognitive function in the chronic phases of TBI have also been observed [24, 25]. For example, we found that over a quarter of patients with a history of moderate-to-severe TBI showed significant declines in cognitive functioning from 1 to 2 years following moderate-to-severe TBI [24].

Our understanding of the presence and neuropathology of this progressive deterioration is growing [12, 13, 15, 18, 22–27]. However, there is a limited understanding of the prognostic factors for deterioration. From a clinical point of view, this understanding is critically needed. Identifying prognostic factors would help to identify those most vulnerable to decline. Identifying “post-injury” prognostic factors, such as mood or activity level after injury, is of particular importance because they may be modifiable, thereby opening opportunities for the prevention or mitigation of degeneration. In contrast, pre-injury prognostic factors, such as age and education, are not modifiable, but knowing which contribute to decline may aid clinical management.

This systematic review will be performed to understand the pre-injury and post-injury prognostic factors for neurodegeneration in moderate-to-severe TBI as an important first step towards informing future intervention research and development.

## Methods/design

### Protocol design and registration

This systematic review protocol was reported as per reporting guidance from the Preferred Reporting Items for Systematic Reviews and Meta-Analysis (PRISMA) guidelines [28]^,^ and registered with the International Prospective Register of Systematic Reviews (PROSPERO; registration ID CRD42019122389).

### Eligibility criteria

Study eligibility will be assessed using a PICOTS framework [29], as detailed below.

#### Population

The review will focus on cohort and cross-sectional studies of adults with moderate-to-severe TBI, with injury severity commonly defined by one of two measures of alterations in consciousness: the Glasgow Coma Scale (GCS) and/or duration of post-traumatic amnesia (PTA). The review will exclude studies that exclusively focus on individuals with mild TBI or studies that include patients with mild, moderate, and severe TBI without disaggregation of results by injury severity. Non-human and/or post-mortem studies will not be included in this review.

#### Index prognostic factor

Prognostic factors can be organized into pre-injury, injury-related, and post-injury variables. More specifically, pre-injury prognostic factors include demographics (such as sex, age, and socioeconomic status), as well as medical history and/or presence of co-morbidities, vocational status, and level of education. Injury-related prognostic factors can include injury severity, cause of injury (e.g., fall, assault), and type of acute care management. Post-injury prognostic factors can include type and amount of therapy received, length of stay in acute care and rehabilitation settings, development of co-morbidities secondary to injury, and level of cognitive and physical activity.

#### Comparator prognostic factors

The goals of this systematic review are to identify all potential prognostic factors for neurodegeneration following TBI and to summarize the effect of said factors. There is no comparator prognostic factor included in our study, as our goal is not to benchmark the effect of a given prognostic factor against another. Instead, the goals are to identify which factors have prognostic value with respect to neurodegeneration following TBI and to summarize the effect of all of these factors.

#### Outcome

The primary outcome will be neurodegeneration, defined as regional or whole-brain reductions in brain volume or white matter integrity, as measured using structural or diffusion-based neuroimaging, respectively [13, 14].

### Information sources

The studies will be retrieved from 6 databases: MEDLINE (OVID interface, 1946 onwards), EMBASE (OVID interface, 1947 onwards), PsychINFO (OVID interface, 1806 onwards), CINAHL (EBSCO interface, 1937 onwards), SportDiscus (EBSCO interface, 1800 onwards), and Cochrane Central Register of Controlled Trials. Articles will be electronically retrieved and in cases where electronic copies are unavailable, hard copies of these articles will be requested from the university library.

### Search strategy

Two members (BS and LM) will develop the search strategy (under the guidance of a health sciences librarian). The search strategy will include terms related to neurodegeneration and traumatic brain injury, using keywords and controlled descriptors (such as MeSH terms, CINAHL headings, PsycINFO thesaurus). The search terms were combined using the Boolean operators ‘AND’ and ‘OR’. A sample search strategy has been included below.

- Search Strategy for Medline (OVID interface, 1946-Present)

1. (volum* loss adj2 (brain* or cereb* or white matter* or grey matter* or gray matter*)).ti,ab,kw.
2. (neuro* adj1 (degener* or deterior* or loss*)).ti,ab,kw.
3. (ventricul* adj1 enlarge*).ti,ab,kw.
4. (atrophy adj2 (brain* or cerebr* or neuro*)).ti,ab,kw.
5. (axon* adj1 (degen* or neurodegen* or damag*)).ti,ab,kw.
6. neurodegenerat*.ti,ab,kw.
7. demyelin*.ti,ab,kw.
8. MeSH exp Neurodegenerative Diseases/
9. 1 or 2 or 3 or 4 or 5 or 6 or 7 or 8
10. (head adj1 (injur* or trauma*)).ti,ab,kw.
11. (brain adj1 (injur* or trauma* or damag*)).ti,ab,kw.
12. TBI*.ab,ti,kw.
13. MeSH exp Brain Injuries/
14. 10 or 11 or 12 or 13
15. 9 and 14
16. exp animal/ not human.sh.
17. 15 not 16
18. limit 17 to English language

Initially, a limited search of MEDLINE will be undertaken; this will be followed by an analysis of index terms and the text words contained in the titles and abstracts used to describe each article retrieved through this initial search. These initial findings, as well as input from the consulting health sciences librarian, will be used to build the second search, the results of which will form the pool of all articles to be screened for our systematic review. Once all full-text articles have been identified, their reference lists will be screened, and studies that cite those included in our review will also be examined for inclusion.

Included articles will be restricted to the English language. However, limitations will not be placed on the year of publication. Moreover, weekly auto alerts will be established such that a list of recently published articles from each of the 6 databases will be forwarded to the two reviewers (AC and LM), who will screen titles of these articles and will determine if they will be included in the full-text screening. The purpose of these automated updates will be to ensure that our review is current.

### Data management

Articles retrieved from each database will be exported as .ris files, and then imported into EndNote for collation and removal of duplicates. Articles will then be imported into Abstrackr [30], an online tool that facilitates blind article screening by multiple reviewers. The program includes a web-based annotation tool that allows reviewers (AC and LM) to collaboratively screen citations for relevance. Abstrackr will automatically identify and mark all the articles over which there is conflict (i.e., disagreement between reviewers). These articles will be discussed with another team member (BS) until consensus is reached about inclusion. It should be noted that prior to a formal screening process, a calibration exercise will be undertaken to pilot and refine the screening process.

### Selection process

Using Abstrackr, title/abstract screening will first be performed by two independent reviewers (AC and LM). They will be able to mark articles as follows: include, potentially include, or exclude from full-text screening. When the reviewers disagree, the article will be re-evaluated and, if the disagreement persists, a discussion moderated by a team member (BS) will be used to make a final decision.

The full-text versions of papers retained after the title and abstract screening will be downloaded for full-text review by the same independent reviewers. During the full-text screening, the two reviewers will maintain a record documenting their reasons for excluding the full-text articles. Again, any conflicts regarding article inclusion will first be resolved through discussion between the two reviewers, and then through third party (BS) arbitration as required.

### Data collection process

For data extraction, two reviewers (AC and LM) will independently populate a customized data extraction form created by the other study team members to summarize the data from the included studies. This form will be piloted and amended as necessary prior to engaging in full-text data extraction. As detailed in Table 1, this will include study, population, and key outcome data. A third reviewer (BS) will be engaged in this process as a guarantor of consistency, cross-checking data extraction forms to identify inconsistencies, and resolution of inconsistent approaches through moderated discussion with AC and LM. Corresponding authors will also be contacted for key information or clarification when data are ambiguous or missing from the published study.

**Table 1:** Fields contained in our data extraction form

Extraction category	Fields
Study characteristics	Author
	Title
	Journal
	Year of publication
	Country of publication
	Inclusion criteria
	Exclusion criteria
	Study design
	Sample size [total and subgroups]
	Control variables [if applicable]
	Study quality [grade]
Population characteristics	Mean age of sample [total and subgroups]
	Time post-injury [total and subgroups]
	Sex (% male) [total and subgroups]
	Years of education [total and subgroups]
	Injury severity (GCS, PTA) [total and subgroups]
	Mechanism of injury [total and subgroups]
	Baseline characterization (e.g., IQ, fitness)
	Time elapsed between injury and baseline [total and subgroups]
	Time elapsed between baseline and follow-up [total and subgroups]
Outcomes—primary Independent variables—correlates to neurodegeneration	Medical [including pre-injury medical conditions or post-injury co-morbidities]
	Neuropsychiatric [including pre-injury or post-injury psychiatric symptoms or diagnoses, including substance use]
	Activity-related [including therapy, leisure pursuits, academic/employment activities, social, physical, cognitive (e.g., reading) activities
	Social and environmental variables [including current living and social circumstances]
	Baseline imaging [imaging type, regions assessed, sequence parameters]—both whole-brain and regional changes in volume or white matter integrity will be assessed
	Follow-up imaging [imaging type, regions assessed, sequence parameters]—both whole-brain and regional changes in volume or white matter integrity will be assessed
Outcomes—primary Dependent variables—measures of neurodegeneration	

### Quality assessment

Each reviewer will independently assess the methodological quality of each study at the full-text screening phase. This will be done in accordance with Quality in Prognostic Factor Studies (QUIPS) checklist [29, 31], which is designed specifically to assess the quality of prognostic studies. This checklist examines the risk of bias in prognostic studies in six domains, namely: study participation, study attrition, prognostic factor measurement, outcome measurement, adjustment for other prognostic factors, and statistical analysis and reporting. The QUIPS checklist will be used to identify significant concerns in the methodological approach or rigor of the studies included in our review. These assessments will also be used to guide this review commentary on the strength and quality of published literature on prognostic factors of neurodegeneration following moderate-to-severe TBI.

### Data synthesis

Our expectation is that there will be insufficient data for a meta-analysis. This is due to the considerable heterogeneity in the population, as well as heterogeneity in the timing of neuroimaging and outcome measures used to assess post-injury factors that may correlate with neurodegeneration.

Therefore, heterogeneity will be assessed using chi-square tests and *I*^2^ tests, and a choice to analyze data narratively will be made if either *p* < 0.1 on chi-square or *I*^2^ > 50% are identified. If there is adequate homogeneity across studies, a random-effects meta-analysis will be conducted to estimate odds ratios of neurodegeneration across correlate using the DerSimonian and Laird method [29], adjusting for factors such as age, sex, and time post-injury. If there are sufficient data to perform multiple smaller meta-analyses of more homogeneous studies, these will also be performed as per the method above.

In the case of inadequate homogeneity of the data across studies, a qualitative synthesis of the data will be undertaken to examine prognostic factors of long-term neurodegeneration after TBI. In this scenario, which we expect is more likely, reviewers will narratively synthesize findings in accordance with recent guidelines [32], to examine risk-factors of long-term neurodegeneration after TBI. To the extent that it is possible, data will be stratified according to study design (within or between-group designs), time of neuroimaging, neuroimaging type/regions assessed, and other relevant categories that arise from the review.

### Primary and secondary outcomes

Data extraction forms will contain fields specific to study and population characteristics as well as outcome measures, as below (Table 1).

### Patient and public involvement statement

For the present review, research questions and methods were informed by the authors’ collective experience working with patients requiring rehabilitative care after a moderate-to-severe TBI. No patients were directly involved in the design of this review study.

## Discussion

Despite a compelling body of evidence suggestive of neurodegeneration in the chronic phases of moderate-to-severe TBI, it remains unclear why some patients are more vulnerable to these declines than others. This challenge is in part due to the significant heterogeneity between patients’ pre-injury factors (e.g., demographics and medical history), to differences in the nature and severity of their brain injuries, and to the variability in their environments post-injury (e.g., type and frequency of therapy, level of cognitive, and physical activity).

Without an understanding of the prognostic factors for neurodegeneration in chronic moderate-to-severe TBI, there is limited clinical ability to effectively prognosticate decline or tailor treatment planning at the individual patient level. More importantly, enhanced understanding of prognostic factors for long term degeneration—in particular, post-injury prognostic factors—would offer the opportunity to develop targeted interventions aimed at mitigating declines and improving long-term brain health in this population. As such, this systematic review was performed to understand the pre-injury, injury-related, and post-injury prognostic factors for neurodegeneration in moderate-to-severe TBI. To our knowledge, this would be the first systematic review on correlates of neurodegeneration in chronic moderate-to-severe TBI, and thus an important first step towards filling critical knowledge gaps and informing future intervention research and development.

## Data Availability

The datasets used and/or analyzed during the current study are available from the corresponding author on reasonable request.

## Abbreviations

GCS: Glasgow Coma Scale
PRISMA: Preferred Reporting Items for Systematic Reviews and Meta-Analysis Protocols
PROSPERO: International Prospective Register of Systematic Reviews
PTA: Post-traumatic amnesia
TBI: Traumatic brain injury
